# Lipidated Protein D vaccination elicits humoral and cellular responses and protects mice against challenge with non-typeable *Haemophilus influenzae*

**DOI:** 10.1128/iai.00647-25

**Published:** 2026-03-31

**Authors:** Terri C. Thayer, Andrew Cox, Eduardo Gonzalez, Jill Mangiafesto, Lei Xu, Michael Pichichero, Ravinder Kaur

**Affiliations:** 1 Center for Infectious Diseases and Immunology, Rochester General Hospital Research Institute 551254, Rochester, New York, USA; University of Pennsylvania Perelman School of Medicine Microbiology, Philadelphia, Pennsylvania, USA

**Keywords:** *Haemophilus influenzae*, Protein D, vaccination, lipoprotein, aluminum hydroxide, ear infection model, nasopharyngeal colonization, acute otitis media, antibody, Th17 cells

## Abstract

Nontypeable *Haemophilus influenzae* (*NTHi*) is a primary cause of acute otitis media (AOM) in children and lower respiratory tract infections in adults. Vaccines that can reduce nasopharyngeal carriage and prevent disease are needed. We used an adult murine model of nasopharyngeal carriage and AOM infection to evaluate immunogenicity and protection provided by subcutaneous and intramuscular vaccination with lipidated Protein D (L-PD) compared with non-lipidated PD (NL-PD) formulated with and without alum adjuvant. ELISA was used for antibody measurements and flow cytometry to characterize T-cell immunity. L-PD + Alum subcutaneous vaccination induced low levels of PD-specific IgG antibodies and significantly elevated Th17 and T effector memory cell activation in cervical lymph nodes and splenocytes compared with NL-PD + Alum. The addition of alum increased bactericidal antibody activity. Intramuscular vaccination with L-PD with and without alum induced 3- to 4-fold log_10_ higher PD-specific IgG antibodies than subcutaneous vaccination but did not elicit Th17 or T effector memory cell activation. Subcutaneous vaccination with L-PD + Alum protected mice within both the nasopharynx and middle ear. Middle ear bacterial density reduction correlated with IgG antibody levels after intramuscular vaccination with L-PD + Alum but not L-PD without alum, yet no protection from nasopharyngeal carriage occurred. PD + Alum vaccination increased humoral and cellular immunity and protected mice from *NTHi* carriage and AOM, whereas NL-PD did not. Activation of Th17 immunity by lipidation of PD and subcutaneous vaccination correlated with protection from nasopharyngeal carriage of *NTHi*.

## INTRODUCTION

Nontypeable *Haemophilus influenzae* (*NTHi*) can cause severe infections in both children and adults, leading to high morbidity and mortality worldwide. *NTHi* has become the most common cause of acute otitis media (AOM) in children (1). It is the primary pathogen isolated in cases of persistent AOM, recurrent AOM, and AOM treatment failures (2–4), and is also a common cause of acute sinusitis and conjunctivitis in children and adults (5–8). Additionally, *NTHi* is a leading cause of acute exacerbations of chronic obstructive pulmonary disease (COPD) in adults (5–8). Invasive disease cases have also been reported to be caused by *NTHi* (9, 10). Moreover, antibiotic resistance to *NTHi* is increasing (11, 12).

Different groups have assessed potential *NTHi* proteins as vaccine candidates, including proteins P4, P6, PD, PE, PF, OMP26, PilA, Hap, HMW, and ZnuA (13–16). We have evaluated lipidated (L) and non-lipidated (NL) formulations of proteins P6, OMP26 alone and fused (17), and NL-Protein D (NL-PD) as vaccine candidates in mouse models (18, 19).

PD, a well-conserved protein from *NTHi,* is included as a carrier protein in a 10-valent pneumococcal conjugate vaccine (PCV10). PCV10 contains eight serotypes conjugated to NL-PD, along with Serotype 18C conjugated to tetanus toxoid and 19F conjugated to diphtheria toxoid. PCV10 produced a 15% reduction in AOM caused by *NTHi* in children (20) but did not reduce nasopharyngeal carriage (21, 22). In our previous study with NL-PD, we found that it reduced the middle ear burden of *NTHi* but had no impact on carriage (19), consistent with the clinical observations in children. Recent clinical studies in adults evaluated *NTHi* proteins D, E, and PilA for safety and immunogenicity (23, 24); however, an efficacy trial in adults failed to show protection from acute exacerbations of COPD (25).

Vaccines targeting respiratory bacteria aim to prevent nasopharyngeal carriage to provide herd immunity and limit contagion risk. Nasopharyngeal carriage is controlled by both antibody transudation and mucosal T-cell responses, particularly Th17-mediated immunity (26–28). Prior work with *Streptococcus pneumoniae* has shown that lipidated protein antigens from pneumococci stimulate T-helper 17 (Th17) cell responses (29). Lipidation of a protein results in the activation of toll-like receptor (TLR) 2 coupled with TLR1 or TLR6, depending upon the triacyl or diacyl lipid nature of the ligands, respectively, and then downstream signaling via NF-κB (30, 31).

Prior work from our laboratory involving *NTHi* proteins OMP26 and P6 found that fusing a lipid to produce L-P6 and L-OMP26 as vaccine constructs resulted in greater stimulation of interleukin (IL)−17A and IL-22 from nasal-associated lymphoid tissue (NALT) in adult mice compared with NL-P6 or NL-OMP26 vaccinations. L-P6 and L-OMP26 vaccination also resulted in higher numbers of memory T cells in NALT (17).

Therefore, in this study, we tested the hypothesis that the addition of a lipid moiety to PD would increase the quantity or quality of PD-specific antibodies and promote Th17 immune responses, resulting in protection from AOM and nasopharyngeal carriage as compared with NL-PD, where an impact on carriage was not observed in our previous work (20). The experiments were designed to advance L-PD as a candidate vaccine and shed light on the past clinical trial of PCV10 in infants, where immunogenicity of NL-PD was observed, resulting in modest protection from AOM but failure to protect against *NTHi* nasopharyngeal carriage.

## MATERIALS AND METHODS

### Generation and purification of lipidated and non-lipidated Protein D

PD is a naturally triacyl lipidated protein; however, PD, as formulated in PCV10, omitted the signal sequence, resulting in NL-PD. To produce L-PD, we used the P4 signal sequence of *NTHi* (as utilized in the Trumenba vaccine) to express L-PD in the periplasm of *E. coli* strain C41 (DE3) (Fig. S1A) and subsequently purified the lipidated protein.

Plasmids (pET-21a) were prepared using the Monarch Plasmid Miniprep Kit (New England Biolabs). A codon-optimized DNA sequence for PD expression was cloned into plasmids with the T7 promoter. Plasmids were co-transformed into chemically competent *E. coli* C41 (DE3) PlysS cells (Lucigen) according to the manufacturer’s protocol. To produce NL-PD, a plasmid lacking the P4 signal sequence was transformed into BL21* *E. coli* cells for cytoplasmic expression as reported previously (19). Colonies were selected using 100 µg/mL ampicillin or 100 µg/mL ampicillin plus 34 µg/mL chloramphenicol and verified by PCR to confirm successful transformation.

### Lipidated protein expression, extraction, and purification

Chemically competent *E. coli* strain C41 (DE3) PlysS containing plasmids were cultured overnight in LB media with ampicillin and chloramphenicol. These pre-cultures were inoculated into 1 L M9 minimal media supplemented with the same antibiotics and were grown at 37°C until OD_600_ ~0.6. Protein expression was induced with 1 mM IPTG, followed by overnight growth at 30°C. Cells were harvested by centrifugation (10,000 × *g*, 10 min), and pellets were stored at −20°C.

Frozen cells were resuspended in lysis buffer (50 mM Tris, 300 mM NaCl, 100 µg/mL lysozyme, 1 mM PMSF) and lysed by sonication. Lysates were incubated at 37°C for 20–30 min, then ultracentrifuged (29,400 × *g*, 30 min, 4°C). Cell pellets containing target protein were extracted with a solution containing 1% Triton X-100, 2% zwittergent 3-14, 50 mM Tris, and 300 mM NaCl, including DNase treatment and sonication, followed by ultracentrifugation (111,000 × *g*, 30 min, 4°C). The extraction process was repeated, and the combined supernatants containing lipidated proteins were collected.

Purification was performed using Bio-Rad Profinity IMAC Ni-charged resin (1 mL resin per liter culture). Resin was equilibrated with 5 mM imidazole and 0.5% zwittergent 3-14. Extracted proteins were incubated with resin for 15–30 min at room temperature. Resin was washed thrice with equilibration buffer (5 mM imidazole/0.5% zwittergent 3-14), and proteins were eluted with 250 mM and subsequently 500 mM imidazole in 0.5% zwittergent 3-14. Eluates were combined, concentrated using a 3 kDa cutoff concentrator (Merck-Millipore; Amicon Ultra-15), and buffer exchanged into 1× TBS with 0.05% zwittergent 3-14. Endotoxins were removed using a high-capacity resin (Thermo/Pierce), and protein concentration was quantified by Micro-BCA assay. Purity of PD was checked via SDS-PAGE gel and stained with SimplyBlue SafeStain (Invitrogen) (Fig. S1B). Purified proteins were stored at −80°C. Purification of non-lipidated Protein D has been described previously (19).

### TLR stimulation specificity

HEK-Blue hTLR2-TLR1 cells (Invivogen) were used to determine triacyl lipidation status, and hTLR2-TLR6 reporter cells (Invivogen) were used to determine diacyl lipidation status as previously described (17). The addition of purified L-PD (1 µg/mL) induced higher stimulation of HEK-Blue hTLR2-TLR1 cells compared with HEK-Blue hTLR2-TLR6 cells, indicating predominant triacyl lipidation of L-PD (Fig. S1C and D, respectively). As expected, NL-PD (1 µg/mL) failed to stimulate either cell line (Fig. S1C and D).

### Mice

C57BL/6J mice were purchased from The Jackson Laboratory and bred in-house. An equal proportion of age-matched (6–8 weeks) male and female mice were used in the study. Mice were used in accordance with US law and NIH regulations, and according to approved guidelines of the Rochester General Hospital Animal Care and Use Committee.

### Vaccines and vaccine schedule

Vaccines consisted of 10 µg L-PD or NL-PD with or without 200 µg aluminum hydroxide (Alhydrogel, Invivogen) formulated in Tris-buffered saline containing 0.05% zwittergent 3-14. Mice were anesthetized with isoflurane (Baxter) and vaccinated subcutaneously at the base of the neck with 100-µL total volume or intramuscular injection in the hind flank (50 µL per thigh per animal) at weeks 0, 1, and 3 (Fig. S2). Blood samples were taken 2 weeks after the second and third vaccinations via tail clip, centrifuged at 6,000 × *g* for 30 min, and serum was collected.

### Influenza virus and *NTHi* co-infection model

We modified the influenza virus and *NTHi* co-infection model from our previous reports (17, 19) to improve reproducibility, reduce the inflammatory impact of the influenza virus strain, and more closely mimic the timing of human-host pathogenesis. Two weeks post-final vaccination, mice were anesthetized with 4% isoflurane in oxygen and intranasally inoculated with 1 × 10⁴ EID₅₀ of Influenza A/X31 (H3N2) in 10 µL sterile PBS. Three days later, under anesthesia, mice received a standardized intranasal challenge of approximately 5 × 10^6^ CFU of *NTHi* strain 86-028-NP or 1 × 10^5^ CFU clinical strain 701 (a clinical isolate from our collection) in 10 µL PBS (Fig. S2). *NTHi* 86-028 was cultured overnight in Brain Heart Infusion broth supplemented with 10 µg/mL hemin and 10 µg/mL NAD to an OD_600_ of 0.5 ± 0.05 (~1 × 10^9^ CFU/mL), centrifuged, and resuspended in PBS prior to inoculation. *NTHi* 701 was cultured for 2.5 h in Brain Heart Infusion broth supplemented with 0.1% Laked Horse Blood (Lampire Biological) and 10 µg/mL NAD to an OD_600_ of 0.2 (**~**2 × 10^8^ CFU/mL). Bacteria were resuspended and diluted in PBS prior to intranasal infection of mice.

Four days after *NTHi* infection, blood was collected retro-orbitally for serum preparation under anesthesia. Mice were euthanized by isoflurane anesthesia followed by cervical dislocation. Nasal washes were obtained by retrograde flushing of 300 µL sterile PBS through the nasopharynx. Ear bullae were excised and homogenized in 1 mL sterile PBS. Samples were serially diluted, plated on chocolate agar, and incubated overnight at 37°C with 5% CO_₂_ for colony-forming unit (CFU) enumeration. Cervical lymph nodes (CLNs) and spleens were harvested and processed into single-cell suspensions in DMEM with 10% DMSO and stored in liquid nitrogen for future use.

### Mouse Protein D serum IgG, IgG-1, and IgG-2b ELISAs

Microplates (Microlon medium binding; Greiner Bio-One) were coated overnight at 4°C with L-PD or NL-PD antigen at 1 µg/mL in 50 mM carbonate buffer, pH 9.5. IgG protein concentrations were similar when using L-PD or NL-PD for antibody capture; therefore, NL-PD was used for all samples. One column of the plate was reserved for coating with goat anti-mouse IgG-Fc (Bethyl, cat#A90-131A) at 1 µg/mL for a mouse IgG standard. The coated plates were washed 5× with 1× PBS/0.1% Tween and blocked with 4% skim milk in 1× PBS/0.1% Tween. Mouse sera were diluted 1:50 followed by eight twofold serial dilutions and added to the washed plates. For the standard column, mouse reference serum (Bethyl, cat. RS10-101) was tested at 20 (IgG), 10 (IgG-1), or 8.7 ng/mL (IgG-2b), followed by eight twofold dilutions to be used as a relative quantitative measurement. Primary incubation was for 1 h at room temperature, followed by five more plate washes and addition of HRP-secondary antibody at 1:6,000 IgG, IgG-1, or IgG-2b (Bethyl Laboratories) and incubated for an additional hour. Plates were washed again before adding TMB substrate (KPL) and allowed to develop for 15 min at room temperature, then stopped by the addition of 1 M H_3_PO_4_. Plates were read at 450 nm. PD IgG titers were measured relative to the mouse standard column concentration because no PD-specific standard is available.

### Bactericidal assay

The bactericidal assay was performed according to published protocol with some modification (32) using sera from individually vaccinated mice or pooled samples when quantities were low. Colonies of *NTHi* (clinical strain 575) were cultured in BHI broth to log phase with OD_600_ at ~0.5 and diluted to 1:80,000 in PCMA buffer (0.2mM CaCl_2_, 1mM MgCl_2_, 0.1% BSA, PBS). Serial dilutions of sera were mixed with 5% human complement (Pel-Freeze #34010-10) and then incubated with the diluted bacteria at 37°C for 60 min while rotating. Samples were plated on chocolate agar and incubated overnight at 37°C with 5% CO_2_ for CFU enumeration. The bactericidal titer was defined as the inverse of the highest dilution that led to >50% killing compared with control bacteria without serum.

### *In vitro* activation and flow cytometry

CLN and spleen samples from vaccinated mice were removed from storage and, after thawing, 1 × 10^5^ lymphocytes were stimulated with 1 × 10^5^ CFU/mL heat-inactivated *NTHi* (strain 86-028-NP) or 1 µg L-PD in DMEM supplemented with 10% FBS, 100 U/mL penicillin, 100 µg/mL streptomycin, and 50 µM 2-mercaptoethanol for 5 days. Activated cells were incubated for 6 h with Golgistop (BD Biosciences) prior to labeling for flow cytometry.

Surface markers were labeled with the following antibodies: CD19-APC (Biolegend, 6D5, 2 µg/mL), CD3-BV785 (Biolegend, 145-2C11, 0.1 µg/mL), CD8a-BV480 (Thermo Fisher, 53-6.7, 4 µg/mL), CD4-BV421 (Biolegend, GK1.5, 0.12 µg/mL), CD44-BUV737 (Thermo Fisher, IM7, 0.5 µg/mL), CD62L-APCeFluor780 (Thermo Fisher, MEL-14, 1 µg/mL), CD69-PE-Cy5 (Biolegend, H1.2F3, 2 µg/mL), CD95-BUV563 (BD Biosciences, Jo2, 1 µg/mL), CCR5-BUV615 (BD Biosciences, C34-3448, 1 µg/mL), CCR6-PE-Fire700 (Biolegend, 29-2L17, 1 µg/mL), and CCR7-BV605 (Biolegend, 4B12, 1 µg/mL).

Cells were fixed and permeabilized with Transcription Factor Fixation/Permeabilization kit (eBioscience), following the manufacturer’s directions. Intracellular targets were labeled with the following antibodies: IFNγ-BUV395 (Thermo Fisher, XMG1.2, 2 µg/mL), TNF-PE-Dazzle594 (Biolegend, MP6-XT22, 4 µg/mL), IL17A-AF488 (Biolegend, TC11-18H10.1, 5 µg/mL), IL21-PE-Cy7 (Thermo Fisher, mhalx21, 2 µg/mL), IL22-PerCpCy5.5 (Biolegend, Poly5164, 2 µg/mL) or IL22–PerCPefluor710 (Thermo Fisher, 1H8PWSR, 2 µg/mL), Tbet-RB705 (BD Biosciences, O4-46, 1 µg/mL), RORγt-PE (Thermo Fisher, B2D, 2 µg/mL), and GATA3-BUV661 (Thermo Fisher, TWAJ, 0.5 µg/mL). Spectral flow cytometry was completed using Cytek Aurora, and samples were analyzed with FlowJo v10.10.0 (BD). Gates were established using Fluorescence minus one and unstimulated controls. Background unstimulated percentages were subtracted to determine antigen-specific increases in identified populations.

### Statistical analysis

Ordinary one-way or Welch’s ANOVA with Dunnett’s T3 multiple comparisons test to compare data to controls after log transformation, and nonparametric Spearman correlation and Pearson correlation tests were used. All analyses were performed using GraphPad Prism 10.3.1. Values of *P* < 0.05 were considered statistically significant.

## RESULTS

### Lipidation of PD enhances immunogenicity

Previous reports suggest lipoproteins and liposomal adjuvants administered via the subcutaneous route can generate long-lasting antibody and cellular responses (29, 33–35), including the generation and support of memory Th17 cells (36, 37). We therefore assessed the immunogenicity of L-PD in a predominantly triacyl formulation compared with NL-PD in an adult mouse model of *NTHi* infection.

**Fig 1:**
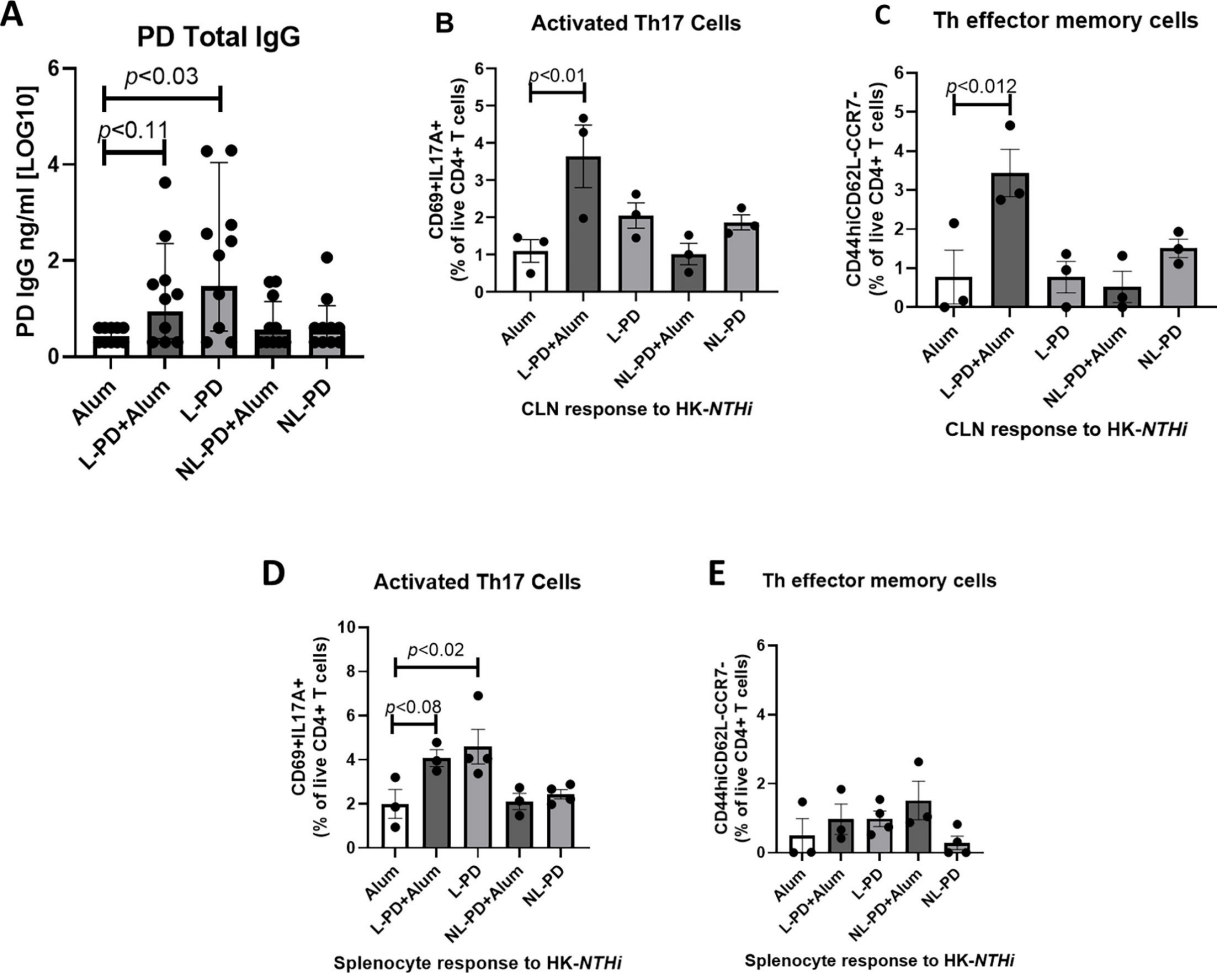
Lipidation of PD enhances immunogenicity. Mice were vaccinated with three doses via subcutaneous injection of alum alone, lipidated (L)-PD + Alum, L-PD, nonlipidated (NL)-PD + Alum, or NL-PD. Mice were challenged with influenza X31, then with *NTHi* 86-028 (5 × 10^6^ CFU) 3 days later. (**A**) Total IgG to PD was measured by ELISA using sera collected from vaccinated mice 4 days after challenge with *NTHi* 86-028. Mean ± SEM of *n* = 5 per group from two independent experiments. Welch’s ANOVA comparison to the control alum group was performed with *P* < 0.05 considered significant. Lymphocytes from (B and C) cervical lymph nodes (CLN) and (D and E) splenocytes of vaccinated and *NTHi* 86-028-challenged mice were stimulated *in vitro* with 1 × 10^5^ CFU/mL heat-killed (HK)-*NTHi* 86-028 for 5 days. Live CD4+ T cells from CLN were analyzed for the expression of (**B**) CD69 and IL-17A to identify activated Th17 cells. (**C**) The percentage of T-helper (Th) effector memory cells (Tem) defined as CD44hiCD62L-CCR7- was compared after stimulation. Live CD4+ T splenocytes were analyzed for the expression of (**D**) CD69 and IL17A to identify activated Th17 cells and (**E**) CD44hiCD62L-CCR7- to identify Tem cells. Mean ± SEM of *n* = 3 is shown. An ordinary one-way ANOVA comparison to the control alum group was performed, with *P* < 0.05 considered significant.

Subcutaneous injection of L-PD + Alum and L-PD without alum resulted in low levels of PD-specific IgG compared with alum control-vaccinated animals (Fig. 1A). NL-PD did not significantly increase antibody, suggesting lipidation improved humoral responses. Lymphocytes from CLN of vaccinated mice were stimulated with heat-killed *NTHi* 86-028 (HK-*NTHi*) to assess cytokine production and activation of CD4^+^ T effector memory cells (Tem) defined as CD44^hi^CD62L^-^CCR7^-^ (Fig. S3). Lymphocytes from CLN of mice subcutaneously vaccinated with L-PD + Alum had significantly increased percentage of activated CD4^+^ T cells that produced IL17 (CD19^-^CD8^-^CD4^+^CD69^+^ IL17+ Fig. S3; Fig. 1B) compared with alum controls. Vaccination with L-PD + Alum also promoted memory responses, as a higher proportion of stimulated CD4^+^ T cells in the CLN were classified as Tem (Fig. 1C). There was no increase in the CLN of Th17 or Tem after vaccination with PD formulations without alum or NL-PD. Stimulated splenocytes also demonstrated increased IL-17 synthesis by T cells from mice vaccinated with L-PD (Fig. 1D), but there was no increase in the Tem population in the periphery (Fig. 1E), suggesting trafficking to the site of infection and draining lymph node.

### Lipidation of PD improves antibody bactericidal function

As subcutaneous vaccination generated low levels of antibody, we sought to determine functional differences associated with the vaccination group. Analyses of IgG subclasses IgG1 and IgG2b did not identify group differences (data not shown). We then assessed the differential functionality of the elicited antibodies through assessment of antibody dilutions able to induce complement-mediated killing of *NTHi*. Bactericidal assays using sera collected from mice subcutaneously vaccinated with L-PD + Alum killed a different clinical strain of *NTHi* (strain 575) at a 1:120 dilution compared to L-PD alone at a 1:60 dilution (Table 1). Non-lipidated vaccine formulations did not generate enough antibody for functional analyses and were not tested. This suggests lipidation and alum together increase antibody-mediated complement killing, improving bactericidal function.

**TABLE 1:** Bactericidal titer after subcutaneous vaccination with L-PD^*a*^

Adjuvant	Pooled sera (*n* = 5 pooled)	Geometric mean (95% CI) of individual mice (*n* = 4)	Median (95% CI) of individual mice (*n* = 4)
Alum	120	145 (4–1,028)	128 (16–1,024)
None	60	63 (22–940)	64 (2–1,024)

^*a*^ Pooled sera shown here is from 5 mice from one experiment to give one bactericidal titer reaction from the pooled sera. The geometric mean and median given are from a different vaccination experiment (*n *= 4), where bactericidal titer was performed on sera from individual mice (*n *= 4).

### Intramuscular vaccination supports humoral but not Th17 immunity

**Fig 2:**
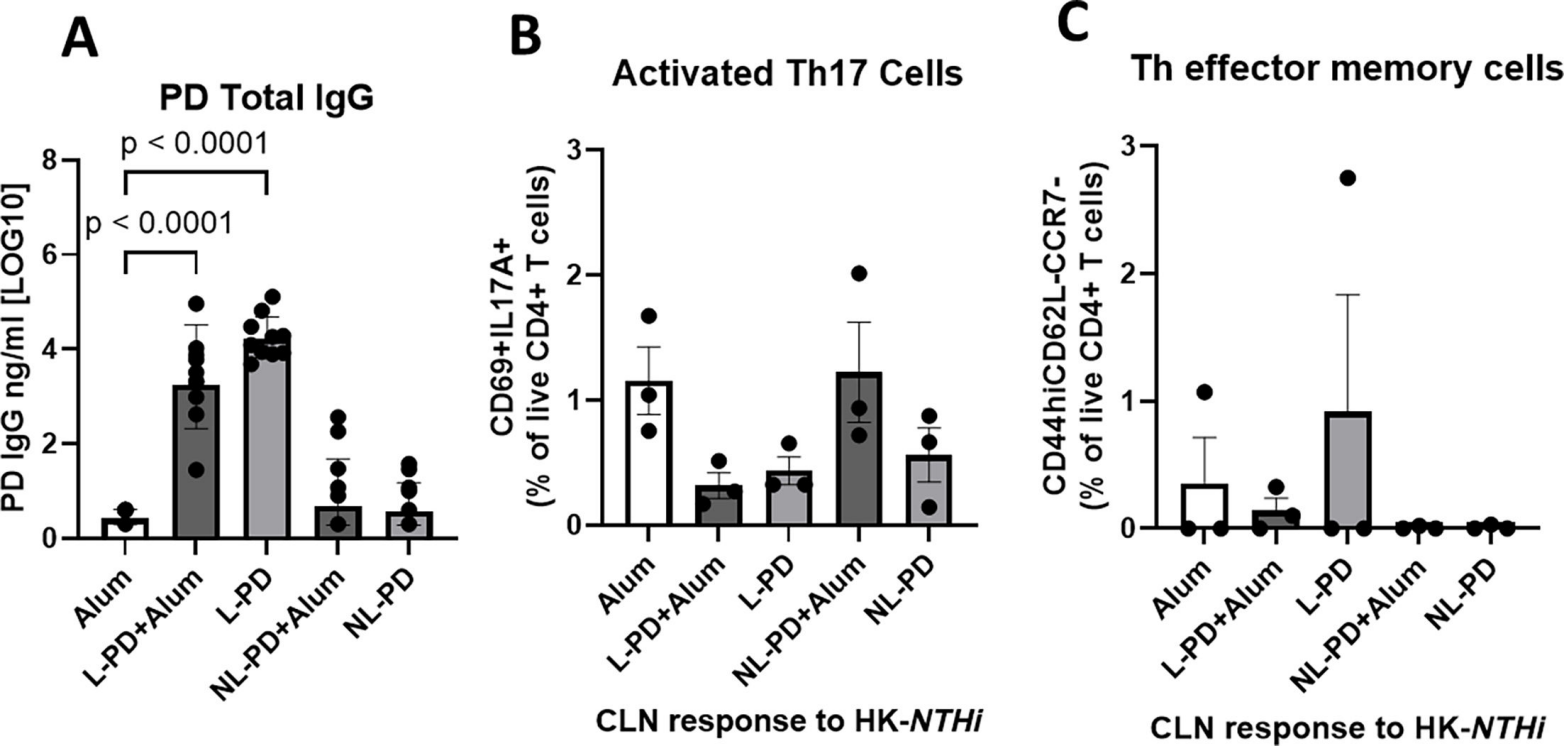
Intramuscular vaccination with L-PD increases antibody production but does not induce Th17 polarization. Mice were vaccinated with three doses via intramuscular injection of alum alone, lipidated (L)-PD + Alum, L-PD, nonlipidated (NL)-PD + Alum, or NL-PD. Mice were challenged with influenza X31, then with *NTHi* 86-028 three days later. (**A**) Total IgG to PD was measured by ELISA in mouse sera collected 4 days post-challenge with *NTHi* 86-028. Mean ± SEM of *n* = 5 per group from two independent experiments. Welch’s ANOVA comparison to the control alum group was performed with *P* < 0.05 considered significant. Lymphocytes from cervical lymph nodes of intramuscularly vaccinated and *NTHi* 86-028-challenged mice were stimulated *in vitro* with 1 × 10^5^ CFU/mL heat-killed (HK)-*NTHi* for 5 days. (**B**) The percentage of live CD4+ that were CD69+IL17A+ and (**C**) CD44hiCD62L-CCR7- was compared after stimulation. Mean ± SEM of *n* = 3 mice per group is shown. An ordinary one-way ANOVA comparison to the control alum group was performed, with *P* < 0.05 considered significant.

We immunized mice by the intramuscular route with L-PD or NL-PD with or without alum. Intramuscular vaccination with L-PD + Alum and L-PD without alum generated 2–3 log_10_ higher levels of PD-specific IgG (Fig. 2A) compared to both alum controls and levels stimulated by subcutaneous vaccination. Intramuscular vaccination with NL-PD + Alum and NL-PD without alum generated low IgG antibody levels. Assessment of cellular immune responses found intramuscular vaccination failed to induce Th17 or Tem populations by stimulated CLN (Fig. 2B and C, respectively) and splenocytes (data not shown). These data suggest lipidation of PD and formulation with an alum adjuvant when delivered intramuscularly does not support Th17 lineage commitment and Tem populations, in contrast to subcutaneous vaccination.

### Subcutaneous vaccination with L-PD and alum reduces *NTHi* burden in the nose and middle ear bullae of adult mice

**Fig 3:**
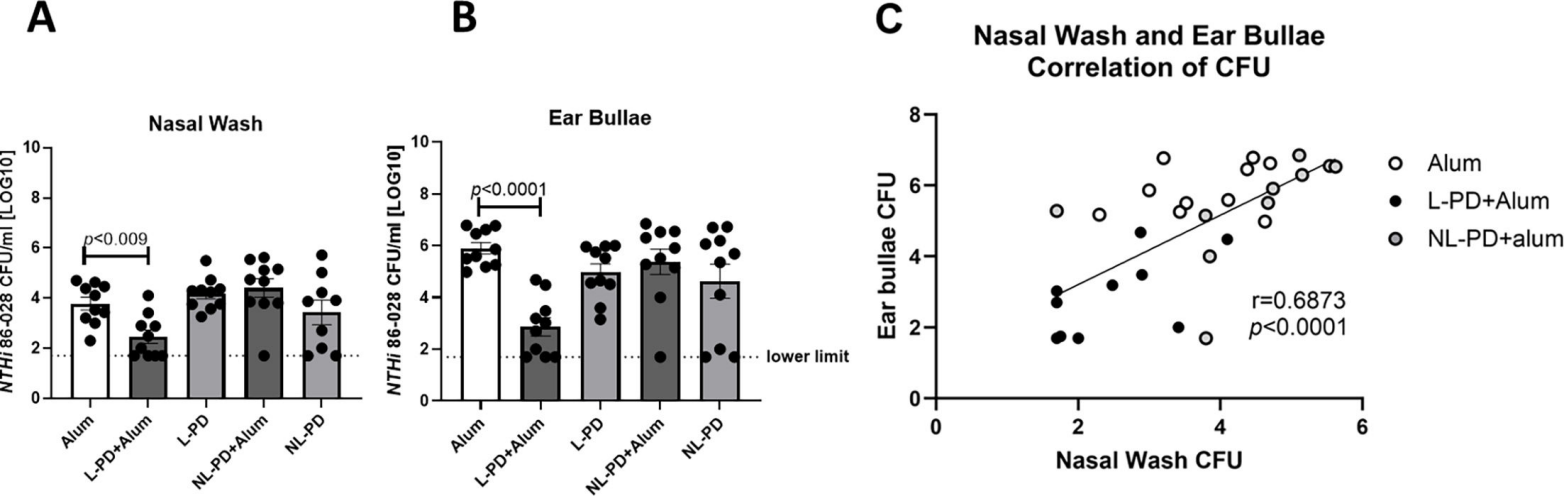
Subcutaneous vaccination with lipidated (L)-PD + Alum reduces bacterial carriage of *NTHi*. Mice were vaccinated with three doses via subcutaneous injection of alum alone, L-PD + Alum, L-PD, nonlipidated (NL)-PD + Alum, or NL-PD. Mice were challenged with influenza X31, then with *NTHi* 86-028 three days later. CFU in the (**A**) nasal wash and (**B**) ear bullae were assessed 4 days post-*NTHi* challenge. Mean ± SEM of *n* = 5 per group from two independent experiments with *NTHi* 86-028 is shown. Welch’s ANOVA comparison to the control alum group was performed with values *P* < 0.05 considered significant. (**C**) CFU in the nasal wash and ear bullae for each mouse receiving subcutaneous Alum (*n* = 10), L-PD + Alum (*n* = 10), or NL-PD + Alum (*n* = 10). Linear Regression, Pearson Correlation, and *P* values were calculated with *P* < 0.05 considered significant.

**Fig 4:**
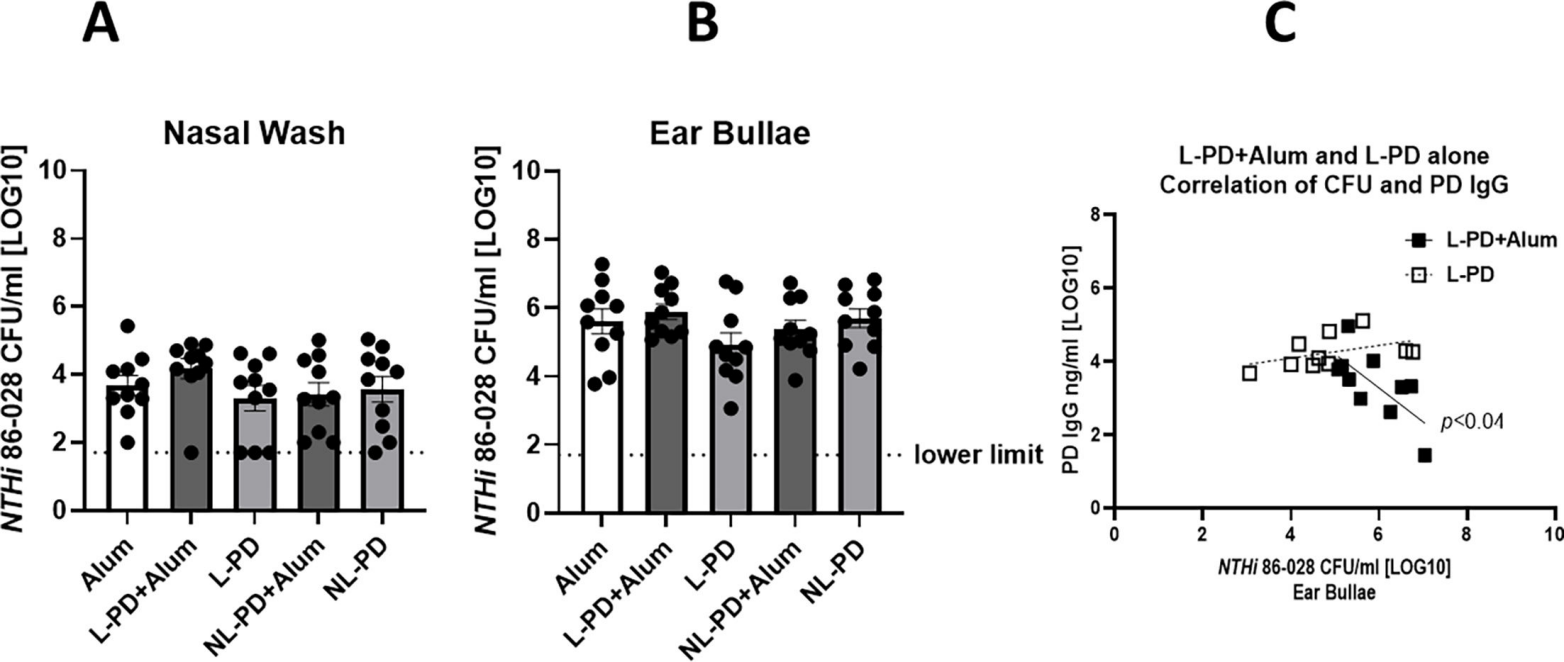
Intramuscular vaccination with lipidated-PD does not protect adult mice from *NTHi* carriage. Mice were vaccinated with three doses via intramuscular injection of Alum alone, L-PD + Alum, L-PD, nonlipidated (NL)-PD + Alum, or NL-PD and then challenged with *NTHi* 86-028. Four days post-challenge, the CFU in the (**A**) nasal wash and (**B**) ear bullae was assessed. Mean ± SEM of *n* = 5 per group from two independent experiments is shown. Welch’s ANOVA comparison to the control alum group was performed with *P* < 0.05 considered significant. (**C**) CFU of ear bullae and PD IgG titers for each mouse (*n* = 10) receiving intramuscular L-PD + Alum (closed square) or L-PD (open square). Nonparametric Spearman Correlation and *P* values were calculated, with *P* < 0.05 considered significant.

Following subcutaneous vaccination, protection from nasopharyngeal carriage and AOM was assessed by enumeration of bacterial burden in nasal wash and ear bullae, respectively. Mice subcutaneously vaccinated with L-PD + Alum had significantly reduced *NTHi* carriage in the nasal wash and ear bullae (Fig. 3A and B, respectively). A correlation of CFU in the nasal wash and ear bullae (Fig. 3C) demonstrates that decreased CFU was observed in both compartments in protected mice. In contrast, administration of L-PD without alum, NL-PD + Alum, and NL-PD without alum had no significant impact on bacterial carriage, suggesting that both lipidation and inclusion of alum are required for establishing protective immunity. The same protection experiments were repeated with a different *NTHi* strain (701), and protection was observed in the middle ear bullae (Fig. 4B) but not in the nasopharynx (Fig. S4A).

Intramuscular vaccination with L-PD + Alum or without alum did not significantly reduce *NTHi* 86-028 burden present in the nasal passage or ears (Fig. 4A and B, respectively). However, an inverse correlation of IgG levels and CFU in the middle ear bullae of L-PD + Alum-vaccinated mice was observed (Fig. 4C), suggesting the antibody generated, while correlative, was insufficient for protection.

## DISCUSSION

In the present study, we used an adult mouse model to study two vaccine constructs of protein D of *NTHi*, L-PD, and NL-PD, formulated with and without alum. We found that biochemical addition of a triacyl lipid to protein D to create L-PD enhanced humoral immunity as well as Th17 and Tem responses when formulated with alum and injected subcutaneously compared to formulation without alum and to non-lipidated protein D. L-PD + Alum vaccination also produced antibodies with better bactericidal activity than vaccination with L-PD without alum. In *NTHi* challenge experiments, we found that L-PD + Alum reduced *NTHi* bacterial density in the nasopharynx and the middle ear bullae during an AOM infection. Intramuscular vaccination with L-PD induced high levels of PD-specific IgG, but this delivery route failed to generate Th17 or Tem cells in response to *NTHi*. Intramuscular vaccination also did not reduce bacterial burden in our challenge model. Our data highlight the contributions of lipidation, adjuvant, and delivery route in priming and directing the subsequent immune response to a specific antigen.

Our finding that L-PD vaccination improved immunogenicity compared to NL-PD, characterized by elevated IgG antibody levels and generation of Th17 and Tem populations, confirmed our hypothesis and was consistent with prior investigations. Elevations in Th17 and Tem immunity were associated with protection from nasopharyngeal bacterial carriage and middle ear infection, advancing L-PD as a vaccine candidate and shedding light on the past clinical trial of PCV10 in infants, where modest protection from AOM but failure to protect against *NTHi* nasopharyngeal carriage was observed. Th17 memory induction is required for protective immunity to many bacterial pathogens, particularly in the mucosa (reviewed in reference [38]). Previous reports found lipidation of pneumococcal proteins increased antigen-presenting cell (APC) synthesis of Th17-skewing cytokines IL-6 and IL-23 compared to non-lipidated proteins (39), and the efficacy of the pneumococcal whole-cell vaccine is dependent upon lipoprotein stimulation of TLR2 and Th17 immunity (40). Through the activation of TLR and the localized cytokine milieu, Th17 lineage commitment has been observed in models of bacterial infection, including *Mycobacterium tuberculosis* (41)*, Pseudomonas aeruginosa* (42)*,* and *Klebsiella pneumoniae* (43). We have previously characterized the immunogenicity of OMP26 and P6, a naturally non-lipidated protein and lipidated protein from *NTHi,* respectively, with lipidation enhancing adaptive immune responses compared to non-lipidated proteins (17). Taken together with the current study, this suggests that modifying the lipidation status of target proteins enhances immunogenicity of vaccines.

Inclusion of an alum adjuvant in addition to lipidation was found to be beneficial for promoting adaptive immune responses. We detected enhanced bactericidal activity induced by L-PD + Alum that correlated with increased protection. Bactericidal antibodies are defined as functional serum antibodies that promote bacterial clearance through enhanced opsonophagocytosis or complement-mediated killing. Previously, we demonstrated bactericidal activity against PD in the natural antibody response of children with AOM (32). Other studies have similarly shown that bactericidal antibodies contribute to protection against AOM caused by *NTHi* (44, 45). Defining the role of bactericidal antibodies specific to PD vaccine antigens highlights their potential as components of novel vaccine strategies to protect against *NTHi* infections.

We found that protective immunity and reduced bacterial carriage were dependent on the route of vaccination. Our results are consistent with other reports characterizing the generation of long-term antibody and Th17 memory responses when lipoproteins are given subcutaneously but not via intramuscular vaccination (36, 41). Subcutaneous vaccination with L-PD + Alum induced T-cell synthesis of IL-17A upon reactivation that correlated with reduced CFU in both the nasopharynx and the ear bullae. This was not observed with intramuscular vaccination, suggesting the localization and processing of antigen directs T-helper lineage polarization in the draining lymph node of subcutaneous vaccination. Our data also suggest memory cells had successfully trafficked to the site of infection in bacterial-challenged mice, as Tem were enriched in the draining lymph node in mice vaccinated subcutaneously with L-PD + Alum but not after intramuscular vaccination. Antigen processing of vaccine lipoproteins likely differs by the route of immunization. The local population of APCs within the skin, including Langerhans cells and dermal DCs, promotes varying T-helper responses (46). The cell-type localization and preference for antigen, along with sensitivity to specific adjuvants, are critical components to skewing T-helper responses and establishing long-term protective immunity. The antigen depots created in the subcutaneous tissue, not present in the muscular tissue, may provide sustained exposure to the antigen needed to establish prolonged Th17 memory. These findings suggest that subcutaneous delivery of lipidated Protein D formulations may represent a viable strategy for enhancing immune activation and durability, informing the rational design of future human vaccines targeting *H. influenzae*.

Vaccines containing lipids can display increased reactogenicity. A meningococcal vaccine that includes a lipidated protein (Trumenba) caused fever in 70% of vaccinated infants, and its administration consequently is not approved for this age group. However, multiple whole-cell vaccine constructs do include lipids and are safely administered to infants worldwide. Therefore, lipids are not contraindicated in infant vaccines. We previously reported that construction of diacyl lipid constructs, as compared to triacyl constructs, correlated with lower pro-inflammatory cytokine IL-6 in the blood of vaccinated adult mice (47). We did not construct a diacyl PD vaccine for comparisons with the experiments reported here. Further work investigating the diacylation versus triacylation status of lipidated protein vaccine constructs and their relative immunogenicity, cellular immune response elicited, and potential reactogenicity is planned in future experiments to understand the parameters for T-cell polarization and establishing protective memory.

In conclusion, our studies support the strategy of lipidation to improve vaccine immunogenicity. Lipidated PD stimulates Th17-mediated immunity to *NTHi* and prevents both nasopharyngeal carriage and middle ear infection. Our observations demonstrating the polarization of Th17 cells and establishment of Tem in animals protected from challenge are noteworthy for translational application to humans for this vaccine construct. It also emphasizes the need to consider the delivery of antigen in local tissue and draining lymph nodes and the role of adjuvant.

## SUPPLEMENTAL MATERIAL

Fig. S1 iai.00647-25-s0001.tif Generation and purification of L-PD and NL-PD.

Fig. S2 iai.00647-25-s0002.tif Vaccination schedule and NTHi/Influenza co-infection model.

Fig. S3 iai.00647-25-s0003.tif Gating strategy of in vitro activated cervical lymph nodes and splenocytes.

Fig. S4 iai.00647-25-s0004.tif Subcutaneous vaccination with L-PD+Alum reduces NTHi 701 bacterial carriage.

ASM does not own the copyrights to Supplemental Material that may be linked to, or accessed through, an article. The authors have granted ASM a non-exclusive, world-wide license to publish the Supplemental Material files. Please contact the corresponding author directly for reuse.
