# Supplementary figures and images for: Lipidated Protein D vaccination elicits humoral and cellular responses and protects mice against challenge with non-typeable Haemophilus influenzae
Source: Infect Immun. 2026 Mar 31;94(5):e00647-25. doi: 10.1128/iai.00647-25 (PMC13163197; doi:10.1128/iai.00647-25)

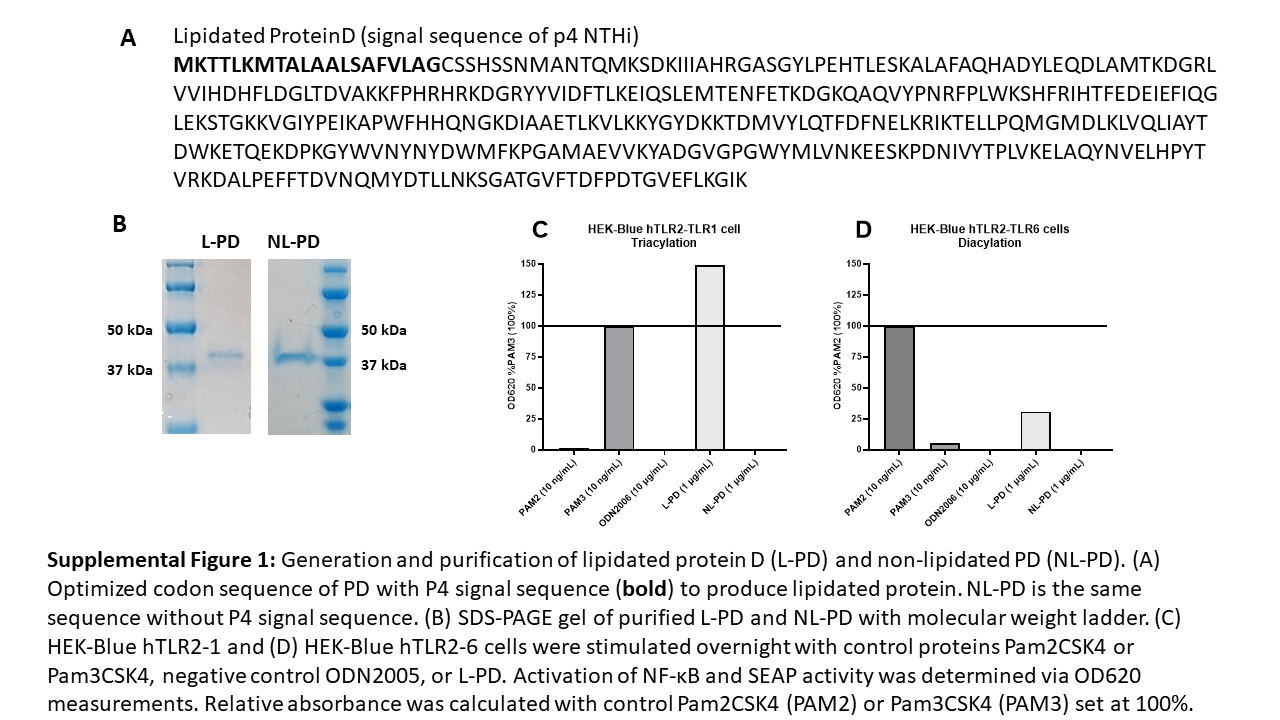

Supplement: Fig. S1 — Generation and purification of L-PD and NL-PD. [file iai.00647-25-s0001.tif]

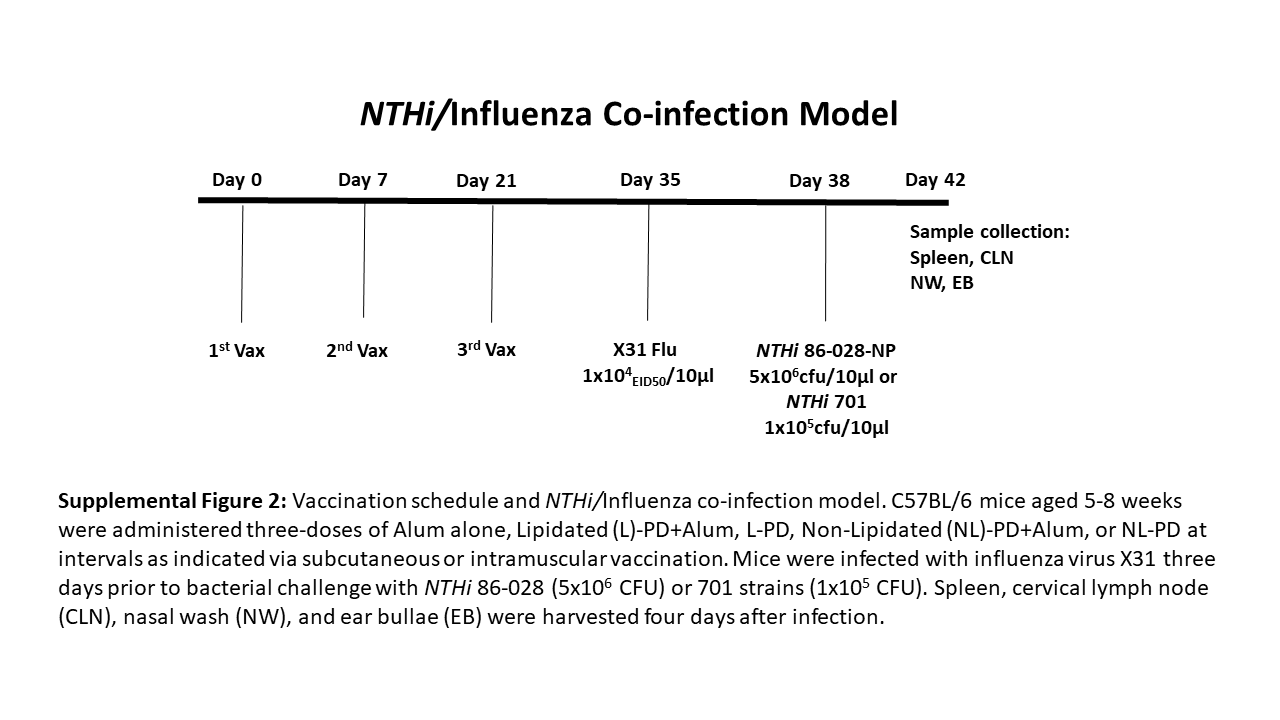

Supplement: Fig. S2 — Vaccination schedule and NTHi/Influenza co-infection model. [file iai.00647-25-s0002.tif]

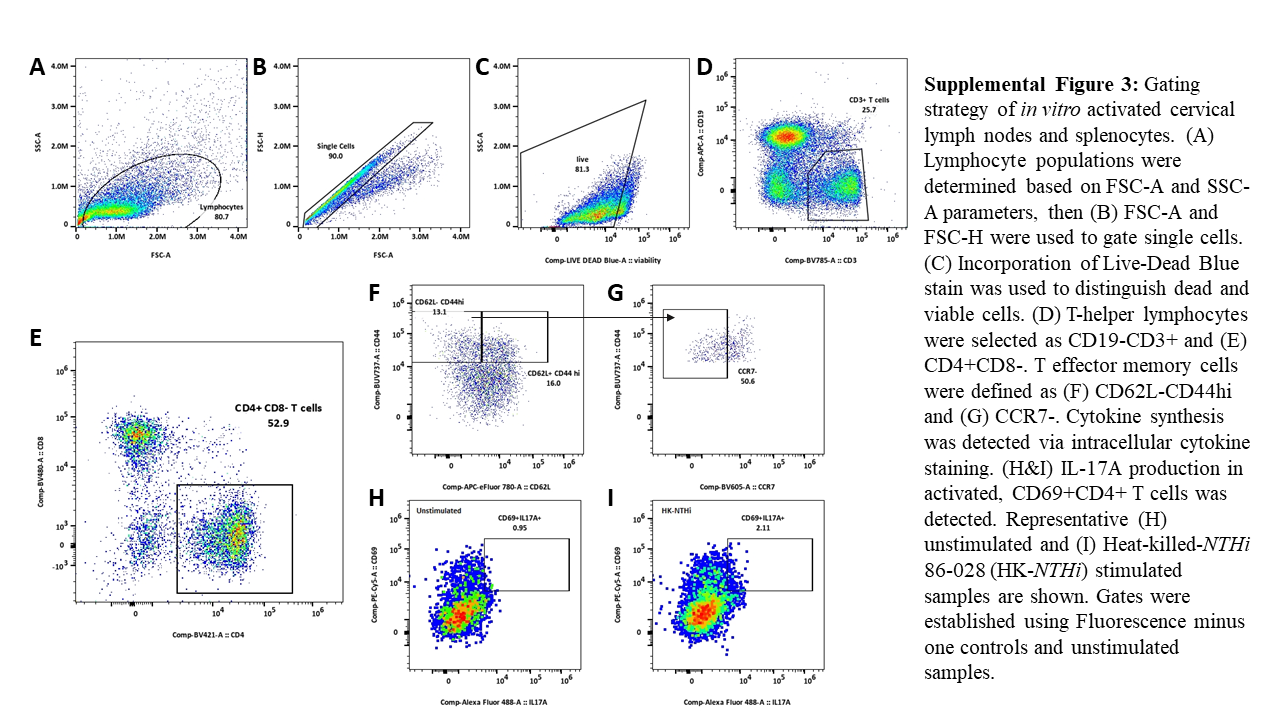

Supplement: Fig. S3 — Gating strategy of in vitro activated cervical lymph nodes and splenocytes. [file iai.00647-25-s0003.tif]

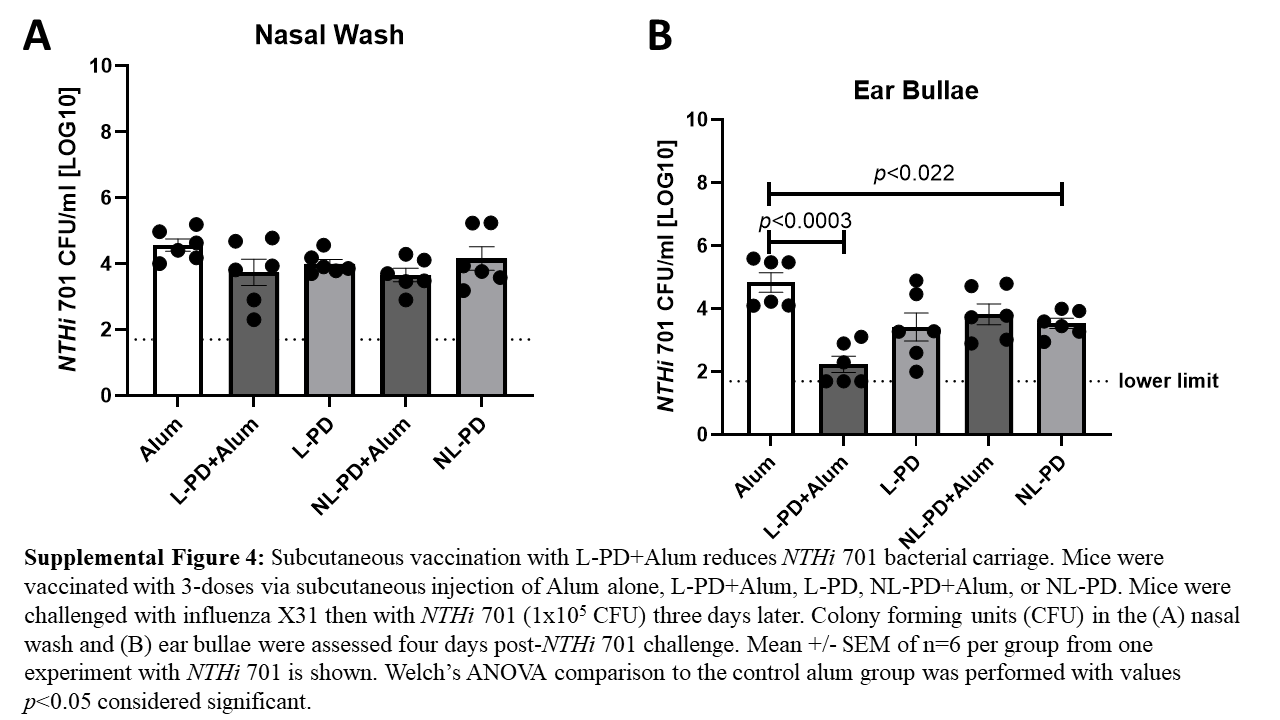

Supplement: Fig. S4 — Subcutaneous vaccination with L-PD+Alum reduces NTHi 701 bacterial carriage. [file iai.00647-25-s0004.tif]
